# Editorial: Salivary biomarkers for oral and systemic diseases

**DOI:** 10.3389/fdmed.2024.1429305

**Published:** 2024-06-12

**Authors:** Mario Taba, Melissa M. Grant

**Affiliations:** ^1^ Department of Oral, Maxillofacial Surgery, and Periodontology, Faculty of Dentistry of Ribeirão Preto, University of São Paulo, Ribeirão Preto, Brazil; ^2^ School of Dentistry, College of Medical and Dental Sciences, University of Birmingham, Birmingham, United Kingdom

**Keywords:** saliva, diagnosis, systemic disease, oral health, biomarkers

**Editorial on the Research Topic** Salivary biomarkers for oral and systemic diseases

We would like to present the research topic “*Salivary biomarkers for oral and systemic diseases*” and the accompanying contributing articles. In this editorial, we aim to highlight the findings of these articles to address this important research area. The topic of salivary biomarkers has become increasingly relevant in recent years, as it has the potential to improve the accuracy and speed of disease diagnosis. We hope that our editorial will offer insight into this field and encourage further research and advances in this area. Thank you for your attention.

Saliva is a readily available and abundant biofluid that can be collected through non-invasive methods. It holds immense potential for monitoring the health and disease of the oral cavity and, potentially, the rest of the body. Saliva contains a diverse range of molecules that can serve as biomarkers, making it a valuable resource for detecting various conditions such as periodontitis, caries, cancer and COVID-19. High-content “omic” approaches have proven to be essential for identifying and discovering the many biomarkers present in saliva. These approaches have been crucial for developing the next generation of biomarkers, as it is likely that a combination of biomarkers will provide a better indication of changes in health than a single biomarker.

Initially, we would suggest the readers explore the narrative review of Basic and Dahlén. In this manuscript, it is shown how microbial interaction is dynamic showing the importance of microbial metabolites in the pathogenesis of periodontal diseases. The authors describe how complex metabolic pathways of bacteria give rise to a cascade of metabolites, including short-chain fatty acids (SCFAs; formic, acetic, propionic, butyric, and valeric acid), amines (indole, scatole, cadaverine, putrescine, spermine, spermidine), and gases (NH3, CO, NO, H2S, H2). The continuous metabolic fluctuations balanced by the inflammatory response create a homeostatic condition between the colonizers and the host response that can be monitored in saliva. Although the mechanisms behind the tissue destruction are still poorly understood, studies addressing the functions of the microbiota, the metabolites, and how they interplay with host tissues and cells, are therefore warranted.

Going in-depth into the biofilm formation, Enax et al. stressed the importance of the dental pellicle by presenting a comprehensive description of its composition and properties. The capacity of the dental pellicle as a layer that protects our teeth from acid attacks and how it plays a crucial role in the remineralization process is detailed. In this regard, it acts as a binding site and source of nutrients for bacteria, while also serving as the foundation for dental plaque formation and saliva components. Through the use of cutting-edge analytic techniques, it is presented a deeper understanding of the pellicle's composition, which consists of a variety of amino acids, proteins, and proteolytic protein fragments. By leveraging this knowledge, the protective properties of the dental pellicle may be modulated to contribute to achieving healthier and stronger teeth.

Periodontitis, a severe gum disease that can lead to tooth loss and other health issues, requires accurate diagnosis and monitoring. However, the current biomarkers have limitations that make them unreliable. In recent years, salivary biomarkers have emerged as a promising alternative. Therefore, Rocha et al. aimed to assess the concentration profile of salivary biomarkers and their diagnostic efficacy for periodontitis activity. Their results demonstrated the consistency and reliability of salivary analytes as biomarkers for periodontal disease, particularly IL-6. These findings have positive implications for the diagnosis and monitoring of periodontitis and may contribute to more accurate and effective treatment outcomes.

During COVID-19 wearing a face mask has become a necessary safety measure. With this in mind, Lee et al. investigated the oral microbiome and the impact of mask-wearing and xerostomia on oral bacteria. The study analyzed the oral bacterial species of 55 generally healthy adults, including *Porphyromonas gingivalis*, *Lactobacillus casei*, *Tannerella forsythia*, and *Treponema denticola*, on the mask's inner surface and in unstimulated and stimulated saliva samples. The results showed that oral bacteria migrated onto the inside of the mask and it was observed a significant correlation between salivary bacteria and oral bacteria on the mask. On the inner surface of the mask, *P. gingivalis* was the most abundant species. These findings emphasize the importance of keeping the mask as clean as possible to reduce the potential bacterial side effects of mask-wearing and highlight the need for good oral hygiene to support overall health. Also, it demonstrates masks as a potential refuge for contaminants confirming the importance of mask-wearing for COVID prevention and control of disease spreading.

The exploration of saliva as a diagnostic tool has been a significant advancement in medical science, offering a non-invasive and cost-effective method for disease detection and monitoring. Saliva contains a wide array of biomolecules, including proteins, nucleic acids, and hormones, reflecting the body's health status. Despite its promise, the integration of saliva diagnostics into clinical practice faces challenges, such as the variability in salivary flow and biomolecule concentrations. The development of saliva-based biosensors is promising, aiming to facilitate point-of-care testing for systemic diseases, which could revolutionize the field by enabling early detection and monitoring with non-invasive methods. These improvements reflect a broader trend towards more patient-friendly diagnostic procedures that do not compromise accuracy or reliability. In summary, saliva has the potential to become a first-line diagnostic sample of choice owing to advancements in detection technologies and combinations of biomolecules with clinical relevance.

